# Draft Genome Sequence of *Nereida* sp. Strain MMG025, Isolated from Giant Kelp

**DOI:** 10.1128/mra.00122-22

**Published:** 2022-05-09

**Authors:** Amanda T. Alker, Natalie A. Hern, Munira A. Ali, Melissa I. Baez, Brianna C. Baswell, Bryn I. Baxter, Alyssa Blitz, Theresa M. Calimlim, Cierra A. Chevalier, Claudia A. Eguia, Tania Esparza, Alaina E. Fuller, Caitlin J. Gwynn, Allison L. Hedin, Ronnesha A. Johnson, Maninder Kaur, Rio T. Laxina, Kouta Lee, Payton N. Maguire, Isabella F. Martelino, Jennifer A. Melendez, Jeannine J. Navarro, Jazmin N. Navarro, James M. Osborn, Mariana R. Padilla, Nicole D. Peralta, John Lawrence R. Pureza, Jesse J. Rojas, Taelor R. Romo, Morsal Sakha, Geronimo J. Salcedo, Kaiden A. Sims, Thanh Ha Trieu, Ingrid R. Niesman, Nicholas J. Shikuma

**Affiliations:** a Department of Biology and Viral Information Institute, San Diego State University, San Diego, California, USA; Montana State University

## Abstract

Here, we report the draft genome sequence of *Nereida* sp. strain MMG025, isolated from the surface of giant kelp and assembled and analyzed by undergraduate students participating in a marine microbial genomics course. A genomic comparison suggests that MMG025 is a novel species, providing a resource for future microbiology and biotechnology investigations.

## ANNOUNCEMENT

**FIG 1:**
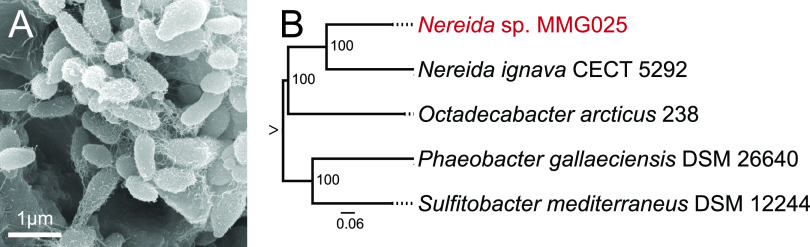
(A) Scanning electron micrograph of *Nereida* sp. MMG025. Bacteria were fixed onto coverslips with 2.5% glutaraldehyde, 4% paraformaldehyde, 0.15% Alcian blue, 0.0075% lysine, and 0.1 M cacodylate buffer. The coverslips were dehydrated and critical point dried prior to coating with 6 nm platinum. Images were obtained on an FEI Quanta 450 variable pressure SEM. (B) Maximum likelihood phylogeny constructed using the codon tree method through PATRIC with 100 single-copy genes and proteins identified using cross-genus families (PGfams) (10, 21–27). The phylogeny root is indicated by an arrow for clarity. The GenBank accession numbers of the sequences used in this analysis are as follows: CVPC00000000 (Nereida ignava CECT 5292), CP003744 (Octadecabacter arcticus 238), CP006967 (Phaeobacter gallaeciensis DSM 26640), and QBKU00000000 (Sulfitobacter mediterraneus DSM 12244).

To engage undergraduates in discovery-based research, novel marine bacteria were isolated and cultured and their genomes sequenced, assembled, annotated, and analyzed by students in a marine microbial genomics (MMG) course at San Diego State University. Strain MMG025 was isolated from the surface of a giant kelp, Macrocystis pyrifera, from the La Jolla Tide Pools, CA, USA (32.8411°N, 117.2817°W), using a sterile cotton swab. A single colony was obtained on marine agar 2216 (BD Difco, Franklin Lakes, NJ, USA) and incubated at 28°C for 72 h. The colonies were transferred to marine broth 2216 and incubated for 72 h at 25°C before storage, DNA isolation, and imaging by scanning electron microscopy (SEM) (Fig. 1).

Genomic DNA was extracted using a Quick-DNA fungal/bacterial miniprep kit (Zymo Research, Irvine, CA, USA). Using 16S rRNA gene amplification with the primers 27F-1492R (1) and Sanger sequencing (Eton Biosciences, San Diego, CA, USA), the closest strain was identified as Nereida ignava CECT 5292 (identity, 97.99%; E value, 0.0). DNA was submitted to the Microbial Genome Sequencing Center (Pittsburgh, PA, USA) for library preparation (Illumina DNA prep kit; San Diego, CA, USA) and whole-genome sequencing (NextSeq 550 platform; Illumina), producing 2 × 150-bp paired-end reads. The reads were trimmed using Trim Galore v0.6.5 (2), assembled using Unicycler v0.4.8 (3) integrated in PATRIC v3.6.12 (4), and annotated using the NCBI Prokaryotic Genome Annotation Pipeline (PGAP) v5.1 (5) with default parameters. MMG025 has a 3.1-Mb genome, a total GC content of 56% with 40 contigs at 242.493× coverage, and an *N*_50_ value of 628,545 bp, with 3,260 predicted coding sequences. Default parameters were used except where otherwise noted.

A phylogenetic analysis revealed that strain MMG025 falls into the genus *Nereida* (Fig. 1), which is part of the *Roseobacter* group, in the family *Rhodobacteraceae* and class *Alphaproteobacteria* (6–9). Comparing strain MMG025 with Nereida ignava CECT 5292 yields an average nucleotide identity (ANI) value of 72.47% (4, 10, 11), a distance that is below the 95% threshold that delineates species (12), suggesting that MMG025 is a new species. We designate the current isolate *Nereida* sp. strain MMG025.

Species within and related to the *Nereida* genus have been found in association with diverse marine eukaryotes. Nereida ignava CECT 5292 is related to an uncultured gall symbiont from red algae (7). Related species from the genus *Octadecabacter* were isolated from an ascidian or compose 70 to 80% of the microbiome of a brittle star (13, 14). *Roseobacter* species are associated with algae and reef-building corals, where they are thought to play important roles in global sulfur cycling, in part through the degradation of dimethylsulfoniopropionate (DMSP) (15). We found that strain MMG025 harbors a homolog of the DMSP demethylase gene *dmdA* (identity, 80%; query coverage, 100%; E value, 0) (16). Because of their natural occurrence with plants and animals and antagonistic properties against pathogenic bacteria, *Roseobacter* species are promising candidates for use as probiotics in aquaculture or for environmental restoration (17–20). The isolation and genome sequence of *Nereida* sp. MMG025 provides a valuable resource for studying the ecology of *Roseobacter* bacteria and serves as an asset for biotechnology applications.

### Data availability.

The genome sequencing and assembly project for strain MMG025 has been deposited at DDBJ/EMBL/GenBank under BioProject accession number PRJNA716944, the raw sequencing data under SRA accession number SRR17607627, and the whole-genome sequence under GenBank accession number JAKFZN000000000.

## References

[1] Weisburg WG, Barns SM, Pelletier DA, Lane DJ. 1991. 16S ribosomal DNA amplification for phylogenetic study. J Bacteriol 173:697–703. doi:10.1128/jb.173.2.697-703.1991. 1987160 PMC207061

[2] Krueger F. 2015. Trim Galore: a wrapper tool around Cutadapt and FastQC to consistently apply quality and adapter trimming to FastQ files. https://www.bioinformatics.babraham.ac.uk/projects/trim_galore/.

[3] Wick RR, Judd LM, Gorrie CL, Holt KE. 2017. Unicycler: resolving bacterial genome assemblies from short and long sequencing reads. PLoS Comput Biol 13:e1005595. doi:10.1371/journal.pcbi.1005595. 28594827 PMC5481147

[4] Davis JJ, Wattam AR, Aziz RK, Brettin T, Butler R, Butler RM, Chlenski P, Conrad N, Dickerman A, Dietrich EM, Gabbard JL, Gerdes S, Guard A, Kenyon RW, Machi D, Mao C, Murphy-Olson D, Nguyen M, Nordberg EK, Olsen GJ, Olson RD, Overbeek JC, Overbeek R, Parrello B, Pusch GD, Shukla M, Thomas C, Vanoeffelen M, Vonstein V, Warren AS, Xia F, Xie D, Yoo H, Stevens R. 2020. The PATRIC Bioinformatics Resource Center: expanding data and analysis capabilities. Nucleic Acids Res 48:D606–D612. doi:10.1093/nar/gkz943. 31667520 PMC7145515

[5] Tatusova T, DiCuccio M, Badretdin A, Chetvernin V, Nawrocki EP, Zaslavsky L, Lomsadze A, Pruitt KD, Borodovsky M, Ostell J. 2016. NCBI Prokaryotic Genome Annotation Pipeline. Nucleic Acids Res 44:6614–6624. doi:10.1093/nar/gkw569. 27342282 PMC5001611

[6] Arahal DR, Pujalte MJ, Rodrigo-Torres L. 2016. Draft genomic sequence of Nereida ignava CECT 5292^T^, a marine bacterium of the family Rhodobacteraceae. Stand Genomic Sci 11:21. doi:10.1186/s40793-016-0141-2. 26929790 PMC4770636

[7] Pujalte MJ, Macián MC, Arahal DR, Ludwig W, Schleifer KH, Garay E. 2005. Nereida ignava gen. nov., sp. nov., a novel aerobic marine α-proteobacterium that is closely related to uncultured Prionitis (alga) gall symbionts. Int J Syst Evol Microbiol 55:631–636. doi:10.1099/ijs.0.63442-0. 15774635

[8] Wagner-Döbler I, Biebl H. 2006. Environmental biology of the marine Roseobacter lineage. Annu Rev Microbiol 60:255–280. doi:10.1146/annurev.micro.60.080805.142115. 16719716

[9] Buchan A, González JM, Moran MA. 2005. Overview of the marine Roseobacter lineage. Appl Environ Microbiol 71:5665–5677. doi:10.1128/AEM.71.10.5665-5677.2005. 16204474 PMC1265941

[10] Ondov BD, Treangen TJ, Melsted P, Mallonee AB, Bergman NH, Koren S, Phillippy AM. 2016. Mash: fast genome and metagenome distance estimation using MinHash. Genome Biol 17:132. doi:10.1186/s13059-016-0997-x. 27323842 PMC4915045

[11] Yoon S-H, Ha S-M, Kwon S, Lim J, Kim Y, Seo H, Chun J. 2017. Introducing EzBioCloud: a taxonomically united database of 16S rRNA gene sequences and whole-genome assemblies. Int J Syst Evol Microbiol 67:1613–1617. doi:10.1099/ijsem.0.001755. 28005526 PMC5563544

[12] Thompson CC, Chimetto L, Edwards RA, Swings J, Stackebrandt E, Thompson FL. 2013. Microbial genomic taxonomy. BMC Genomics 14:913. doi:10.1186/1471-2164-14-913. 24365132 PMC3879651

[13] Kim Y-O, Park I-S, Park S, Nam B-H, Park J-M, Kim D-G, Yoon J-H. 2016. Octadecabacter ascidiaceicola sp. nov., isolated from a sea squirt (Halocynthia roretzi). Int J Syst Evol Microbiol 66:296–301. doi:10.1099/ijsem.0.000715. 26508418

[14] Morrow KM, Tedford AR, Pankey MS, Lesser MP. 2018. A member of the Roseobacter clade, Octadecabacter sp., is the dominant symbiont in the brittle star Amphipholis squamata. FEMS Microbiol Ecol 94:30. doi:10.1093/femsec/fiy030. 29471328

[15] Raina JB, Dinsdale EA, Willis BL, Bourne DG. 2010. Do the organic sulfur compounds DMSP and DMS drive coral microbial associations? Trends Microbiol 18:101–108. doi:10.1016/j.tim.2009.12.002. 20045332

[16] Howard EC, Sun S, Biers EJ, Moran MA. 2008. Abundant and diverse bacteria involved in DMSP degradation in marine surface waters. Environ Microbiol 10:2397–2410. doi:10.1111/j.1462-2920.2008.01665.x. 18510552

[17] Sonnenschein EC, Jimenez G, Castex M, Gram L. 2021. The Roseobacter-group bacterium Phaeobacter as a safe probiotic solution for aquaculture. Appl Environ Microbiol 87:e0258120. doi:10.1128/AEM.02581-20. 33310713 PMC8090895

[18] Damjanovic K, Blackall LL, Webster NS, van Oppen MJH. 2017. The contribution of microbial biotechnology to mitigating coral reef degradation. Microb Biotechnol 10:1236–1243. doi:10.1111/1751-7915.12769. 28696067 PMC5609283

[19] Egan S, Harder T, Burke C, Steinberg P, Kjelleberg S, Thomas T. 2013. The seaweed holobiont: understanding seaweed-bacteria interactions. FEMS Microbiol Rev 37:462–476. doi:10.1111/1574-6976.12011. 23157386

[20] Peixoto RS, Rosado PM, de Leite DCA, Rosado AS, Bourne DG. 2017. Beneficial microorganisms for corals (BMC): proposed mechanisms for coral health and resilience. Front Microbiol 8:341. doi:10.3389/fmicb.2017.00341. 28326066 PMC5339234

[21] Stamatakis A. 2014. RAxML version 8: a tool for phylogenetic analysis and post-analysis of large phylogenies. Bioinformatics 30:1312–1313. doi:10.1093/bioinformatics/btu033. 24451623 PMC3998144

[22] Wattam AR, Davis JJ, Assaf R, Boisvert S, Brettin T, Bun C, Conrad N, Dietrich EM, Disz T, Gabbard JL, Gerdes S, Henry CS, Kenyon RW, Machi D, Mao C, Nordberg EK, Olsen GJ, Murphy-Olson DE, Olson R, Overbeek R, Parrello B, Pusch GD, Shukla M, Vonstein V, Warren A, Xia F, Yoo H, Stevens RL. 2017. Improvements to PATRIC, the all-bacterial bioinformatics database and analysis resource center. Nucleic Acids Res 45:D535–D542. doi:10.1093/nar/gkw1017. 27899627 PMC5210524

[23] Cock PJA, Antao T, Chang JT, Chapman BA, Cox CJ, Dalke A, Friedberg I, Hamelryck T, Kauff F, Wilczynski B, De Hoon MJL. 2009. Biopython: freely available Python tools for computational molecular biology and bioinformatics. Bioinformatics 25:1422–1423. doi:10.1093/bioinformatics/btp163. 19304878 PMC2682512

[24] Davis JJ, Gerdes S, Olsen GJ, Olson R, Pusch GD, Shukla M, Vonstein V, Wattam AR, Yoo H. 2016. PATtyFams: protein families for the microbial genomes in the PATRIC database. Front Microbiol 7:118. doi:10.3389/fmicb.2016.00118. 26903996 PMC4744870

[25] Edgar RC. 2004. MUSCLE: multiple sequence alignment with high accuracy and high throughput. Nucleic Acids Res 32:1792–1797. doi:10.1093/nar/gkh340. 15034147 PMC390337

[26] Stamatakis A, Hoover P, Rougemont J. 2008. A rapid bootstrap algorithm for the RAxML Web servers. Syst Biol 57:758–771. doi:10.1080/10635150802429642. 18853362

[27] Letunic I, Bork P. 2016. Interactive tree of life (iTOL) v3: an online tool for the display and annotation of phylogenetic and other trees. Nucleic Acids Res 44:W242–W245. doi:10.1093/nar/gkw290. 27095192 PMC4987883

